# Biosorption Mechanism of Aqueous Pb^2+^, Cd^2+^, and Ni^2+^ Ions on Extracellular Polymeric Substances (EPS)

**DOI:** 10.1155/2020/8891543

**Published:** 2020-06-22

**Authors:** Di Cui, Chong Tan, Hongna Deng, Xunxue Gu, Shanshan Pi, Ting Chen, Lu Zhou, Ang Li

**Affiliations:** ^1^Pharmaceutical Engineering Technology Research Center, Harbin University of Commerce, Harbin 150076, China; ^2^State Key Laboratory of Urban Water Resource and Environment, School of Environment, Harbin Institute of Technology, Harbin 150090, China

## Abstract

Heavy metal pollution has been a focus with increasing attention, especially Pb^2+^, Cd^2+^, and Ni^2+^ in an aqueous environment. The adsorption capacity and mechanism of extracellular polymeric substances (EPS) from *Agrobacterium tumefaciens* F2 for three heavy metals were investigated in this study. The adsorption efficiency of 94.67%, 94.41%, and 77.95% were achieved for Pb^2+^, Cd^2+^, and Ni^2+^ adsorption on EPS, respectively. The experimental data of adsorption could be well fitted by Langmuir, Freundlich, Dubinin–Radushkevich isotherm models, and pseudo-second-order kinetic model. Model parameters analysis demonstrated the great adsorption efficiency of EPS, especially for Pb^2+^, and chemisorption was the rate-limiting step during the adsorption process. The functional groups of C=O of carboxyl and C-O-C from sugar derivatives in EPS played the major role in the adsorption process judged by FTIR. In addition, 3D-EEM spectra indicated that tyrosine also assisted EPS adsorption for three heavy metals. But EPS from strain F2 used the almost identical adsorption mechanism for three kinds of divalent ions of heavy metals, so the adsorption efficiency difference of Pb^2+^, Cd^2+^, and Ni^2+^ on EPS could be correlated to the inherent characteristics of each heavy metal. This study gave the evidence that EPS has a great application potential as a bioadsorbent in the treatment of heavy metals pollution.

## 1. Introduction

Heavy metal pollution mainly comes from papermaking, smelting, electroplating, and other industrial wastewater and the overuse of pesticide and fertilizer [1]. Heavy metal pollutants are potentially harmful to the environment and human health, and they are not easily degraded by microorganisms in water. People intake heavy metal-contaminated water or food over an extended period, then they will suffer from various diseases or even cancer, such as anemia, bone pain, and chronic respiratory diseases for a long-term exposure to lead, cadmium, and nickel. In general, contaminated water often contains more than one heavy metal, such as industrial effluents, municipal wastewater, and industrial wastewater [2–4]. Therefore, exploring effective methods for controlling heavy metal pollution and improving the water environment, especially for lead, cadmium, and nickel, are necessary.

At present, the most commonly used treatment techniques for heavy metal pollution include chemical precipitation, ion exchange, adsorption, membrane separation, oxidation reduction, and electrochemical [5–22]. Among these methods, adsorption is preferred for its simplicity, efficiency, flexibility in design, low waste production, and environmental-friendly characteristics for certain biosorbents [23]. Recently, microbial extracellular polymeric substances (EPS) have become a popular research topic in the effective treatment of heavy metal pollution due to its safety, efficiency, low energy consumption, and simple operation [24–30].

EPS produced by *Agrobacterium tumefaciens* F2 is a complex compound with high molecular weight and used to adsorb Pb^2+^, Cd^2+^, and Ni^2+^ pollutants in this study. Our previous researches focused on heavy metals or antibiotics adsorbed by bioflocculant MFX, which is one kind of EPS extracted from *Klebsiella* sp. J1 [31–38]. The results showed the great potential of EPS as water treatment materials and guided our subsequent studies. However, the main components of EPS produced by strain F2 are polysaccharide, which is different from the protein as the main component in bioflocculant MFX produced by strain J1. It was still unknown for the application potential of EPS produced by strain F2. Thus, it was used to adsorb heavy metal contaminants, and the adsorption mechanisms were systematically investigated via qualitative and quantitative analyses, thereby providing a new available bioadsorbent in water treatment.

## 2. Experimental Section

### 2.1. Strains and Reagents

*Agrobacterium tumefaciens* F2 is isolated by our group and now deposited in the China Common Microbial Culture Collection (CGMCC No. 10131). Lead nitrate, cadmium chloride, and nickel nitrate were purchased from Sigma-Aldrich, St Louis, MO, USA. Medium components were purchased from the Sinopharm Chemical Reagent Co., Ltd., Shanghai, China. Ultrapure water for all experiments was prepared with the Milli-Q system. All chemicals were analytical grade.

### 2.2. EPS Preparation

Strain F2 was applied to prepare EPS by the fermentation culture. The fermentation medium was composed of the following ingredients (g/L): glucose10, K_2_HPO_4_ 5, KH_2_PO_4_ 2, NaCl 0.1, MgSO_4_•7H_2_O 0.2, yeast extract 0.5, and urea 0.5 adjusted at pH 7.2-7.5. Strain F2 was precultured in the fermentation medium to obtain the seed liquid, which was then inoculated into the fermentation medium with 5% carried by a sterilized fermentor. The relevant culture parameters were set at 30°C, 150 rpm for 24 h with 2.5 L min^−1^. Then, the final fermentation liquid was centrifuged to eliminate the bacteria, and the precooling ethanol was added into the residual supernatant to collect white flocs and then dialyzed for 24 h. The flocs were freeze-dried by vacuum to obtain the dry powder of EPS and dissolved into ultrapure water before use.

### 2.3. Batch Adsorption Experiments

The stock solutions (100 mg L^−1^) of Pb^2+^, Cd^2+^, and Ni^2+^ were prepared by dissolving lead nitrate, cadmium chloride, and nickel nitrate in ultrapure water. Working solutions were obtained by appropriate dilution of the stock solutions with ultrapure water and pH adjustment using 1 mol L^−1^ HNO_3_ or NaOH. In each batch adsorption experiment, 0.2, 0.7, and 0.8 g L^−1^ adsorbents were added into 20 mL of Pb^2+^, Cd^2+^, and Ni^2+^ aqueous solution (20 mg L^−1^, pH 6.0) and stirred for 0–70 min at 30°C. After adsorption, the concentrations of initial and residual ions in the aqueous solution were then measured by inductively coupled plasma optical emission spectrometry (ICP-OES; Optima 5300 DV, PE, USA) with the detection limit of 10 *μ*g L^−1^. All samples were filtered by 0.45 *μ*m cellulose acetate fiber before measurement. The adsorption efficiency (*η*) and the adsorption capacity (*q_e_*) of Pb^2+^, Cd^2+^, and Ni^2+^ on EPS were calculated as follows:

(1) $$\begin{array}{c} qe=\frac{\left(C0-Ce\right)\;V}{M}, \end{array}$$

(2) $$\begin{array}{c} \eta =\frac{\left(C0-Ce\right)}{C0}\times 100\%, \end{array}$$

where *C_0_* and *C_e_* are the initial and equilibrium concentrations of heavy metal ion, respectively (mg L^−1^), *V* is the solution volume (L), and *M* is the used amount of EPS (g). The average values were recorded with standard deviations within ±1.3%, and some error bars are not shown due to the magnitude being smaller than that of the symbols used to plot the graphs.

### 2.4. Adsorption Isotherms and Kinetics

Langmuir, Freundlich, and Dubinin–Radushkevich isotherm models were used to determine the sorption equilibrium at 20°C, 30°C, and 40°C, respectively. To investigate the adsorption isotherm, the initial concentration of heavy metal ions was ranged at 5–50 mg L^−1^, and other conditions were consistent with the abovementioned batch adsorption experiments. For sorption kinetic experiment of heavy metal ions on EPS, the experimental data were analyzed using pseudo-first-order and pseudo-second-order kinetic models. The sorption time was during 2.5–70 min, and other parameters were the same with the abovementioned batch adsorption experiments. All models and key parameters are shown in Table 1.

### 2.5. Characterization of Adsorption Mechanism

The adsorption mechanism of heavy metal ions on EPS and characteristics before and after adsorption was analyzed using Fourier-transform infrared spectroscopy (FTIR), *Zeta* potential analysis, and three-dimensional fluorescence spectrophotometry (3D-EEM) to examine the interactions between EPS and Pb^2+^, Cd^2+^, and Ni^2+^, respectively. EPS loading Pb^2+^, Cd^2+^, and Ni^2+^ samples under the optimal experimental conditions were collected and then rinsed to remove free heavy metal ions using ultrapure water. EPS (before and after Pb^2+^, Cd^2+^, and Ni^2+^ loading) were processed by vacuum freeze-drying. The spectra in the range of 400–4000 cm^−1^ were recorded via an FTIR spectrometer using the KBr disc technique. The *Zeta* potential of the system in the entire process was measured with zeta meter equipment. 3D-EEM was applied to study the variation of active ingredients before and after adsorption via a three-dimensional fluorescence spectrometer (FP6500, JASCO, Japan). Scanning parameters were set as the emission spectra of 220–450 nm at 1 nm increment by varying the excitation wavelength of 220–650 nm at 5 nm increment. A blank solution (Milli-Q water) was subtracted from the sample.

## 3. Results and Discussion

### 3.1. Adsorption Efficiency of Heavy Metals on EPS

**Figure 1:**
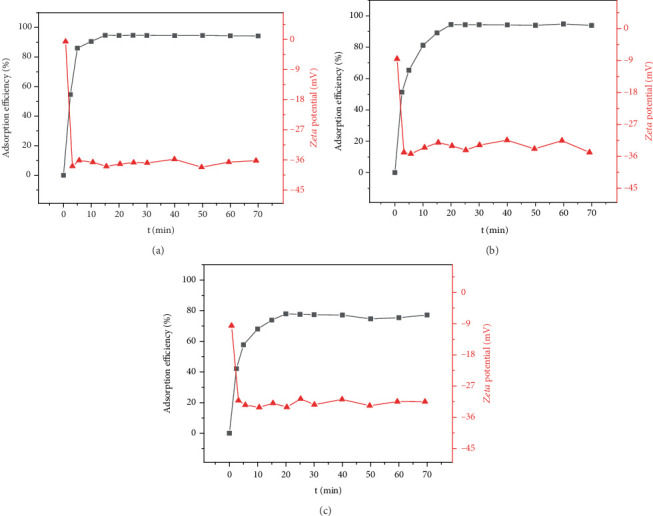
Adsorption efficiency and *Zeta* potential of Pb^2+^ (a), Cd^2+^ (b), and Ni^2+^ (c) adsorption on EPS.

Figure 1 shows the adsorption efficiency and *Zeta* potential of metal ions on EPS at different adsorption time. The adsorption efficiency increased rapidly in the initial 5 min and increased gradually until adsorption saturation at almost 20 min with the highest adsorption efficiency of 94.67%, 94.41%, and 77.95% for Pb^2+^, Cd^2+^, and Ni^2+^ on EPS. Thus, EPS exhibited superior adsorption efficiency for target pollutants, especially Pb^2+^ and Cd^2+^. However, the adsorption efficiency for Ni^2+^ on EPS is clearly not as ideal as Pb^2+^ and Cd^2+^, so the further adsorption mechanism is still needed to explain the adsorption difference. *Zeta* potential analysis was used to analyze the stability of adsorption reaction along with different time before and after Pb^2+^, Cd^2+^, and Ni^2+^ adsorption on EPS. As seen in Figure 1(b), the *Zeta* potentials of reaction system rapidly decreased after adding EPS into Pb^2+^, Cd^2+^, and Ni^2+^ and reached stable at -37.90, -34.9, and -31.2 mV, respectively. Subsequently, the *Zeta* potential remained stable along with the increased adsorption efficiency, thus indicating that the whole adsorption reaction process is stable. Negatively charged EPS was favorable for its adsorption for positively charged heavy metals, so it exhibited the superior adsorption efficiency for Pb^2+^, Cd^2+^, and Ni^2+^.

### 3.2. Isotherm Models

#### 3.2.1. Langmuir Adsorption Isotherm Model

**Figure 2:**
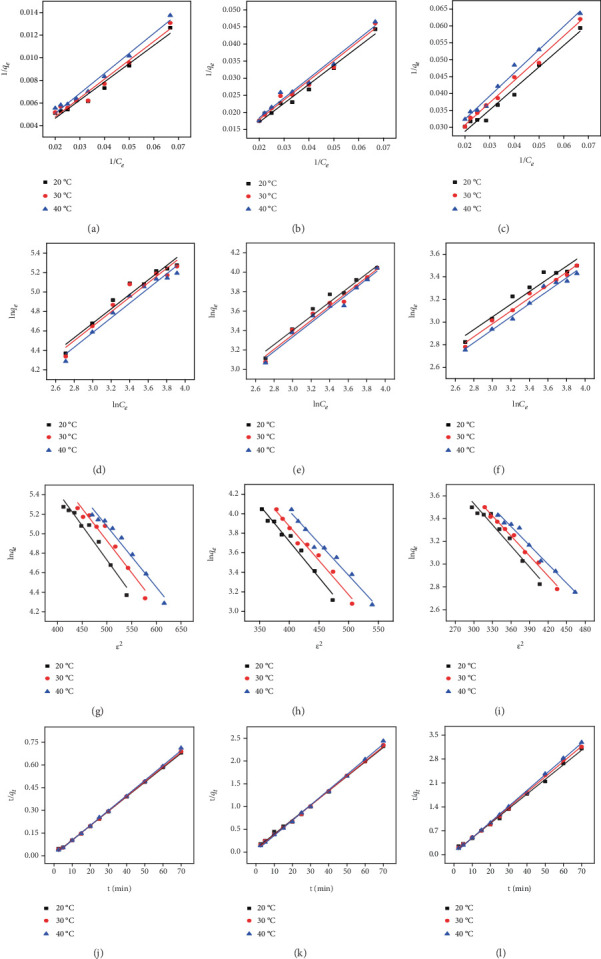
Langmuir (a–c), Freundlich (d–f), Dubinin–Radushkevich (g–i) isotherms, and pseudo-second-order kinetics (j–l) model of Pb^2+^, Cd^2+^, and Ni^2+^adsorption on EPS.

The fitting results of the Langmuir adsorption isotherms of Pb^2+^, Cd^2+^, and Ni^2+^ on EPS at 20°C, 30°C, and 40°C are shown in Figures 2(a)–2(c). The results showed that the *R*^2^ are all greater than 0.90, indicating that Pb^2+^, Cd^2+^, and Ni^2+^ adsorption on EPS could be fitted well by Langmuir adsorption isotherm models. The data for the adsorption process of Pb^2+^, Cd^2+^, and Ni^2+^ on EPS satisfactorily fitted to the Langmuir model in an aquatic system with *R*^2^ > 0.90, indicating that monolayer adsorption could exist [31]. The model parameters are shown in Table 2, in which *q_m_* gradually decreases and *b* increases with the increased temperature, indicating the exothermic nature of the adsorption process.

#### 3.2.2. Freundlich Adsorption Isotherm Model

The fitting results of the Freundlich isotherm model are shown in Figures 2(d)–2(f), and the model parameters are presented in Table 3. The results suggested that adsorption of Pb^2+^, Cd^2+^, and Ni^2+^ on EPS is also consistent with the Freundlich isotherm model with *R*^2^ > 0.90. With the gradual increase of temperature, the gradually decreased *K_F_* of Pb^2+^, Cd^2+^, and Ni^2+^ adsorption on EPS indicated that the adsorption reaction is exothermic [39]. *n* > 1 indicated the good adsorption capacity of Pb^2+^, Cd^2+^, and Ni^2+^ on EPS [31, 37].

#### 3.2.3. Dubinin–Radushkevich Adsorption Isotherm Model

The model is used to judge whether the adsorption process is completed by a physical or chemical reaction [40]. The model parameters of Dubinin–Radushkevich can be used to explain the adsorption process with *R*^2^ > 0.90. The fitting results of Dubinin–Radushkevich models and parameters at 20°C, 30°C, and 40°C are presented in Figures 2(g)–2(i) and Table 4, respectively. Based on the Dubinin–Radushkevich model, the physical adsorption is resulted from Van der Waals forces judged by that *E* value was lower than 8 kJ mol^−1^, whereas the chemical adsorption usually involves ion exchange judged by that the *E* value was 8–16 kJ mol^−1^ [41]. *E* values of Pb^2+^, Cd^2+^, and Ni^2+^ adsorption on EPS are between 8 kJ mol^−1^ and 16 kJ mol^−1^, respectively, indicating that the adsorption process is mainly completed by the chemical adsorption. The above analysis showed that the adsorption process of Pb^2+^, Cd^2+^, and Ni^2+^ on EPS could be well fitted by the Langmuir, Freundlich, and Dubinin–Radushkevich isotherm models (*R*^2^ > 0.90), indicating the complex adsorption process involved in multiple adsorption mechanism, especially chemical adsorption related to ion exchange.

### 3.3. Kinetic Models

The pseudo-first- and second-order kinetic models were applied to fit the data for adsorption behavior. However, the pseudo-first-order dynamic model could not effectively fit the adsorption process with *R*^2^ < 0.80 (data not shown). The pseudo-second-order kinetic model is usually used to clarify the limiting step during the adsorption process. The model was used to analyze the adsorption process and mechanism via quantitative approaches in this study. The fitting results of the pseudo-second-order kinetics model are shown in Figures 2(j)–2(l), and the model parameters are presented in Table 5. *R*^2^ > 0.90 indicated that the adsorption process can be better fitted by the pseudo-second-order kinetic model. The results showed that the chemical adsorption was the rate-limiting step during the adsorption process [24].

The apparent activation energy (*Ea*) is calculated from the reaction rate *k* based on the Arrhenius formula in the pseudo-second-order kinetics. The adsorption process is physical adsorption when the *Ea* is 5–40 kJ mol^−1^ and chemical adsorption when the *Ea* is 40–800 kJ mol^−1^ [32, 42]. The *Ea* values of Pb^2+^, Cd^2+^, and Ni^2+^ adsorption on EPS were 709.27, 660.44, and 472.23 kJ mol^−1^, respectively, indicating a chemical adsorption process.

### 3.4. Adsorption Mechanism

**Figure 3:**
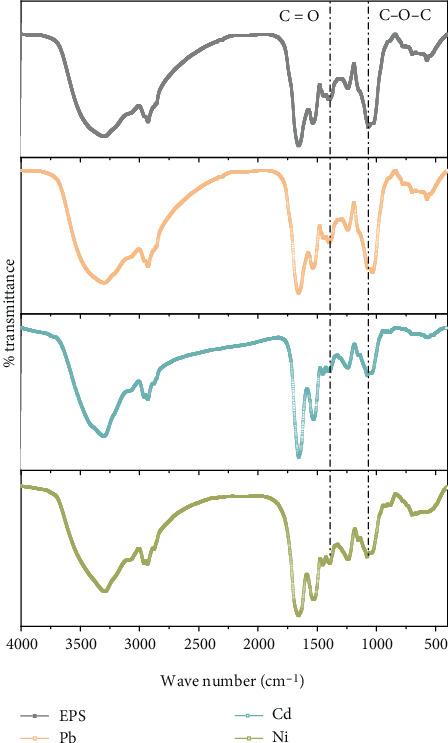
Functional group analysis of EPS before and after Pb^2+^, Cd^2+^, and Ni^2+^ adsorption.

Several studies have shown that the functional group is a key factor for contaminant adsorption on the EPS. The infrared spectra of EPS before and after adsorption of Pb^2+^, Cd^2+^, and Ni^2+^ are shown in Figure 3, and several peaks are observed, including O-H, C=O, N-H, C-N, C-O-C, and C-O in EPS [32, 33]. As shown in Figure 3, obvious changes of the peak intensity in C=O of carboxyl and C-O-C bands from sugar derivatives were observed after heavy metal adsorption. This finding might be explained by the polysaccharides as the main constituent in EPS played the key role during the adsorption process.

**Figure 4:**
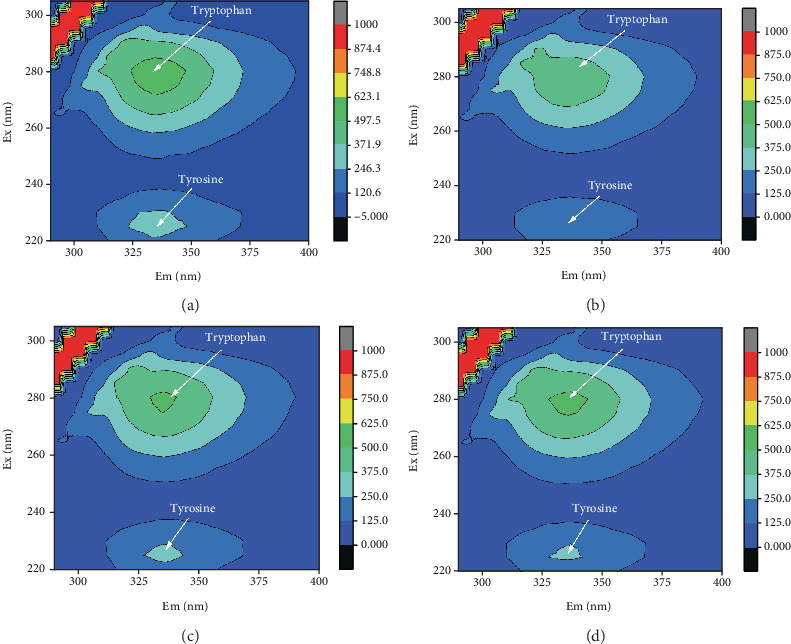
3D-EEM spectrum of EPS (a) before adsorption, and after adsorption of (b) Pb^2+^, (c) Cd^2+^, (d) Ni^2+^.

3D-EEM spectrum exhibited that *λ*ex/*λ*em = (270–280) nm/(325–335) nm and *λ*ex/*λ*em = (225–235) nm/(325–335) nm could represent aromatic amino acid tryptophan and tyrosine of protein-like substances [43].  Figure 4 showed that their fluorescence intensity weakened after EPS absorbing Pb^2+^, Cd^2+^, and Ni^2+^, displaying different levels of quenching. The fluorescence intensity of tyrosine proteins in EPS showed relatively more obvious quenching after absorbing Pb^2+^, Cd^2+^, and Ni^2+^. Results showed the tyrosine of protein-like substances in EPS also played a somewhat role in the adsorption for heavy metals. A possible explanation was that polysaccharide is the main component in EPS produced by strain F2 [44], while the low protein content in EPS resulted in the minor change during the adsorption of heavy metals.

In summary, EPS from strain F2 used the almost identical adsorption mechanism for three kinds of divalent ions of heavy metals. The adsorption efficiency difference of Pb^2+^, Cd^2+^, and Ni^2+^ on EPS could be correlated to the inherent characteristics of each heavy metals, which deserve an in-depth investigation via a quantitative structure–activity relationship (QSAR). The obvious changes in C=O of carboxyl and C-O-C bands from sugar derivatives via FTIR could support the viewpoint of that the polysaccharides as the main constituent in EPS played the key role during the adsorption process of Pb^2+^, Cd^2+^, and Ni^2+^ ions. In addition, the weak quenching changes in tyrosine of protein-like substances in EPS via 3D-EEM was also observed after absorbing heavy metals, which could indicate protein-like substances in EPS also assisted in heavy metals adsorption. At present, EPS has been reported to be used in the Sb(V) reduction and adsorption, which was enhanced through nZVI coating [45]. Therefore, we would consider applying EPS from strain F2 into the redox-adsorption of other substances, such as perchlorate and vanadate [46, 47], in the future work.

## 4. Conclusion

EPS from *Agrobacterium tumefaciens* F2 exhibited effective adsorption efficiency for Pb^2+^, Cd^2+^, and Ni^2+^, especially for Pb^2+^. But EPS from strain F2 used the almost identical adsorption mechanism for three kinds of divalent ions of heavy metals, so the adsorption efficiency difference of Pb^2+^, Cd^2+^, and Ni^2+^ on EPS could be correlated to the inherent characteristics of each heavy metals. Thermodynamics and kinetics analysis displayed the exothermic nature of the adsorption process, the good adsorption capacity of adsorbents, and the key role of chemical adsorption. The adsorption mechanism demonstrated Pb^2+^, Cd^2+^, and Ni^2+^ adsorption on EPS was mainly attributed to the functional groups of the C=O of carboxyl and C-O-C from sugar derivatives. To some extent, amino acid protein-like substances in EPS also assisted in heavy metals adsorption. EPS from strain F2 as a bioadsorbent has great application potential in the treatment of heavy metal ions from contaminated aquatic systems.

## Figures and Tables

**Table 1:** Thermodynamic and kinetics models of heavy metals adsorption on EPS.

Models	Formula	Model parameters
Langmuir adsorption isothermal model	*q*_*e*_ = *q*_*m*_*bC*_*e*_/1 + b*C*_*e*_	*C*_*e*_——the initial concentration of heavy metals (mg L^−1^) *q*_*e*_——the unit adsorption capacity when the initial concentration is Ce (mg g^−1^) *q*_*m*_——maximum unit adsorption capacity (mg g^−1^) *b*——Langmuir adsorption equilibrium constant (L mg^−1^)
Freundlich adsorption isothermal model	*q*_*e*_ = *K*_*F*_*C*_*e*_^1/*n*^	*K*_*F*_——adsorption capacity (mg g^−1^) 1/*n*——Freundlich adsorption capacity
Dubinin–Radushkevich adsorption isothermal model	*q*_*e*_ = *q*_*m*_exp(−*kε*^2^)	*q*_*e*_——equilibrium adsorption capacity (mg g^−1^) *q*_*m*_——maximum unit adsorption capacity (mg g^−1^) *k*——constant related to adsorption capacity (mol^2^/kJ^2^)
	*ε* = *RT*ln(1 + (1/*C*_*e*_))	*R*——ideal gas constant (8.314 J mol^−1^ K^−1^) *T*——thermodynamic temperature *C*_*e*_——initial concentration of contaminants (mg L^−1^) Average adsorption energy *E* = (2 k)^−0.5^
Pseudo-first order kinetics model	log(*q*_*t*_ − *q*_*e*_) = log*q*_*e*_ − (*k*_1_/2.303)*t*	*t*——adsorption time (min); *q*_*t*_——unit adsorption capacity after t min (mg g^−1^); *q*_*e*_——the maximum unit adsorption capacity (mg g^−1^); *k*_1_——pseudo-first-order reaction rate constant
Pseudo-second order kinetics model	*t*/*q*_*t*_ = (1/*k*_2_*q*_*e*_^2^) + (1/*q*_*e*_)*t*	*k*_2_——pseudo-second-order reaction rate constant

**Table 2:** Parameters of Langmuir adsorption isotherms.

Heavy metals	Temperature (°C)	*q_m_* (mg g^−1^)	*b* (L mg^−1^) × 10^−3^	*R* ^2^
Pb^2+^	20	714.29	8.71	0.97
	30	666.67	9.01	0.97
	40	625.00	9.07	0.98
Cd^2+^	20	104.17	20.16	0.96
	30	97.09	21.03	0.96
	40	92.59	21.80	0.96
Ni^2+^	20	51.28	34.20	0.96
	30	48.08	35.48	0.94
	40	45.05	36.53	0.97

**Table 3:** Parameters of Freundlich adsorption isotherms.

Heavy metals	Temperature (°C)	*K_F_* (mg g^−1^)	*n*	*R* ^2^
Pb^2+^	20	11.63	1.3484	0.95
	30	11.22	1.3452	0.94
	40	10.18	1.3259	0.97
Cd^2+^	20	5.05	1.6739	0.93
	30	4.68	1.6464	0.94
	40	4.57	1.6447	0.95
Ni^2+^	20	4.69	2.0080	0.94
	30	4.47	2.0076	0.93
	40	4.25	2.0072	0.97

**Table 4:** Parameters of Dubinin–Radushkevich model.

Heavy metals	Temperature (°C)	*E* (kJ mol^−1^)	*R* ^2^
Pb^2+^	20	8.45	0.96
	30	8.70	0.95
	40	8.91	0.97
Cd^2+^	20	9.05	0.93
	30	9.28	0.95
	40	9.62	0.95
Ni^2+^	20	9.53	0.95
	30	9.90	0.93
	40	10.21	0.97

**Table 5:** Parameters of pseudo-second-order kinetics model.

Heavy metals	Temperature (°C)	*q_m_* (mg g^−1^)	*k*_2_ (min^−1^) × 10^−2^	*R* ^2^
Pb^2+^	20	105.26	1.04	0.99
	30	103.09	1.57	0.99
	40	101.01	3.77	0.99
Cd^2+^	20	31.55	1.33	0.99
	30	30.86	2.09	0.99
	40	29.85	4.40	0.99
Ni^2+^	20	23.42	2.74	0.99
	30	22.42	4.00	0.99
	40	21.65	6.45	0.99

## Data Availability

Data can be available by contacting the corresponding author.
